# A Lattice Boltzmann BGK Model with an Amending Function for Two-Dimensional Second-Order Nonlinear Partial Differential Equations

**DOI:** 10.3390/e27070717

**Published:** 2025-07-02

**Authors:** Xiaohua Bi, Junbo Lei, Demei Li, Lindong Lai, Huilin Lai, Zhipeng Liu

**Affiliations:** 1 School of Liberal Arts and Sciences, North China Institute of Aerospace Engineering, Langfang 065000, China; bixiaohua@nciae.edu.cn; 2 Fujian Key Laboratory of Analytical Mathematics and Applications (FJKLAMA), Center for Applied Mathematics of Fujian Province (FJNU), Key Laboratory of Analytical Mathematics and Applications (Ministry of Education), School of Mathematics and Statistics, Fujian Normal University, Fuzhou 350117, China; 118012021161@student.fjnu.edu.cn (J.L.); dmli079@fjnu.edu.cn (D.L.); 3 Center of Philippine Studies, Fujian Normal University, Fuzhou 350117, China; lindong@fjnu.edu.cn; 4 School of Science, Tianjin Chengjian University, Tianjin 300384, China

**Keywords:** lattice Boltzmann method, second-order nonlinear partial differential equation, Chapman–Enskog expansion, D2Q4 model

## Abstract

A mesoscopic lattice Boltzmann method based on the BGK model is proposed to solve a class of two-dimensional second-order nonlinear partial differential equations by incorporating an amending function. The model provides an efficient and stable framework for simulating initial value problems of second-order nonlinear partial differential equations and is adaptable to various nonlinear systems, including strongly nonlinear cases. The numerical characteristics and evolution patterns of these nonlinear equations are systematically investigated. A D2Q4 lattice model is employed, and the kinetic moment constraints for both local equilibrium and correction distribution functions are derived in the four velocity directions. Explicit analytical expressions for these distribution functions are presented. The model is verified to recover the target macroscopic equations in the continuous limit via Chapman–Enskog analysis. Numerical experiments using exact solutions are performed to assess the model’s accuracy and stability. The results show excellent agreement with exact solutions and demonstrate the model’s robustness in capturing nonlinear dynamics.

## 1. Introduction

The solution of nonlinear partial differential Equations (PDEs) constitutes a core area of research in applied mathematics and computational science, as such equations frequently arise in the modeling of complex physical, biological, and engineering systems [1,2]. Nonlinear PDEs are widely used to describe phenomena such as fluid dynamics, heat transfer, wave propagation, chemical reactions, biological pattern formation, and financial modeling [3,4,5,6]. Due to the intrinsic nonlinearity, these equations often exhibit rich and diverse behaviors, including shock formation, wave interactions, and pattern emergence, which render exact solutions intractable in most cases [2,3]. Consequently, considerable efforts have been devoted to the development of robust and efficient numerical methods, such as finite difference, finite element, spectral, and kinetic-based schemes, for approximating solutions and exploring the qualitative dynamics of nonlinear PDEs under various initial and boundary conditions [7,8]. Due to the fact that nonlinear PDEs only have exact solutions under special initial and boundary conditions and lack a unified expression, it is therefore necessary to vigorously develop efficient and high-precision numerical simulation methods.

In recent years, kinetic theory-based methods have witnessed rapid development. Originally devised to simulate fluid flows, approaches such as the lattice Boltzmann method (LBM) [9,10,11] and the discrete Boltzmann method (DBM) [12,13,14,15,16,17,18,19,20] have also proven effective in solving nonlinear problems. Among them, the LBM has undergone significant advancements, leading to the development of various models tailored to different classes of nonlinear PDEs. By modeling the evolution of particle distribution functions on a discrete lattice, LBM captures nonlinear macroscopic behaviors through local interactions and collision rules. Its inherent simplicity, parallelizability, and flexibility make it well-suited for simulating nonlinear systems with complex geometries and boundary conditions [21]. Compared with the conventional numerical methods, the LBM enjoys many notable advantages, such as geometrical flexibility, numerical efficiency and ease in incorporating complex boundary conditions. Furthermore, it can be naturally adapted to parallel processes computing [22,23].

Many researchers attempted to extend the LBM to solve nonlinear PDEs. Wei et al. (2018) [24,25] introduced a two-dimensional lattice Boltzmann model for thermal incompressible flows. By modifying the equilibrium distribution function and coupling a discrete force, the model improves stability and accuracy. Wang (2020) developed a lattice Boltzmann model to simulate (3+1) D solitary waves in dusty plasma governed by the extended Zakharov–Kuznetsov equation, confirming the model’s validity through numerical experiments [26]. Gorakifard et al. (2022) [27] proposed the meshless local Petrov–Galerkin cumulant LBM to enhance aeroacoustic analysis by improving stability at low viscosities and computational efficiency. Their method integrates cumulant collision modeling and mesh-free streaming, demonstrating superior accuracy and reduced runtime compared to conventional approaches. Boghosian et al. (2024) [28] explore numerical approximations of LBM for inhomogeneous advection in one dimension. Their work innovatively derives high-order equivalent partial differential equations, demonstrating the critical role of initialization in accuracy. Spectral methods validate asymptotic error behavior in steady and unsteady states. Chen et al. (2024) [29] presented a novel fourth-order multiple-relaxation-time LBM for two-dimensional diffusion equations, bridging mesoscopic and macroscopic approaches. By deriving a corresponding finite-difference scheme, they rigorously establish accuracy and unconditional stability, offering valuable theoretical insights and computational advancements in modeling diffusion phenomena. Chai et al. (2016) [30] developed a multiple-relaxation-time lattice Boltzmann model for general nonlinear anisotropic convection–diffusion equations. Their MRT model recovers the correct macroscopic equations and achieves second-order spatial convergence. Compared to the BGK model, the MRT approach reduces numerical slip on boundaries and improves stability by tuning relaxation parameters. Wang et al. (2024) [31] applied the LBM to solve the generalized Zakharov equation, enhancing numerical accuracy and efficiency in plasma wave simulations. Their model demonstrates second-order convergence and superior computational performance compared to traditional methods, advancing nonlinear wave analysis in plasma physics. Wang et al. (2024) [32] applied the lattice Boltzmann method to study wave propagation governed by variable coefficient nonlinear Schrödinger equations, demonstrating advantages in accuracy and efficiency. Liang et al. (2024) [33] proposed a novel thermal lattice Boltzmann model for accurately simulating liquid-vapor phase changes. By avoiding complex gradient and Laplacian calculations, their approach enhances numerical precision while simplifying the modeling process. Chen et al. (2025) [34] proposed a fourth-order multiple-relaxation-time LBM for d-dimensional coupled Burgers’ equations using the Cole–Hopf transformation. This approach eliminates nonlinear convection terms, enhancing stability and accuracy. Chai et al. (2025) [35] proposed a novel mesoscopic regularized LBM-based macroscopic numerical scheme for the Allen–Cahn equation. Li et al. (2025) [36] proposed a novel LBM for a class of viscous wave equations by transforming the original equation to eliminate mixed third-order derivatives. Their method improves numerical stability and achieves second-order accuracy, validated through simulations. Du et al. (2025) constructed a lattice Boltzmann model with BGK operator for solving the fractional Laplacian Reynolds-averaged Navier–Stokes equations by leveraging the fractional centered difference scheme [37]. They also proposed a lattice Boltzmann model with a suitable equilibrium distribution function to solve the surface quasi-geostrophic equations with fractional Laplacian [38].

Apart from successful verification in general problems of fluid mechanics, it has elegant application in relevant domains, ranging from fluid turbulence, multi-phase flows, chemical reactions and hydromagnetics. Recent research has mainly focused on expanding its application areas and improving computational methods for LBM. In applications, Bocanegra et al. (2024) applied LBM to simulate sound wave propagation, demonstrating its excellent performance in handling complex geometric boundaries and the coupling of sound fields with mean flow [39]. Ginzburg (2025) used three-dimensional LBM to simulate the full-volume flow of Newtonian fluids in the colon, contributing to a better understanding of gastrointestinal physiology and pathology [40]. In computational methods, Maquart et al. (2022) developed an LBM version with deformable boundaries, using a Dirichlet velocity scheme to precisely describe surface deformation on regular computational grids without requiring traditional LBM adaptive boundary grids or surface remeshing [41]. This approach is crucial for simulating dynamic shape changes in biological tissues. Hosseini et al. (2024) innovatively proposed a new LBM model and numerical simulation strategy for combustion simulations, making it more stable and efficient in handling compressible flow and multi-component transport problems in combustion simulations [42]. Wawrzyniak et al. (2025) introduced an LBM-based quantum algorithm for solving convection–diffusion equations, providing a new method for solving Navier–Stokes equations on quantum computers in the future [43].

In this paper, we consider two-dimensional second-order nonlinear partial differential Equation (SONPDE) with initial condition. The basic form is shown as follows

(1) $$\left\{\begin{array}{c} \frac{\partial u\left(x,t\right)}{\partial t}+b_{1}\frac{\partial \varphi \left(u\left(x,t\right)\right)}{\partial x}+b_{2}\frac{\partial \varphi \left(u\left(x,t\right)\right)}{\partial y}=\nu \left(\frac{\partial^{2}u\left(x,t\right)}{\partial x^{2}}+\frac{\partial^{2}u\left(x,t\right)}{\partial y^{2}}\right),\\ u\left(x_{L},t\right)=\psi \left(x_{L},t\right),x_{L}\in \partial \Omega ,\\ u\left(x,0\right)=u_{0}\left(x\right), \end{array}\right.$$

where $\Omega \subset R^{2}$ is a bounded region, x=(x,y) ∈Ω, u(x,t) represents the wave displacement at position x and time *t*. φ(u(x,t)) is the known differential functions of u(x,t), used to model nonlinear convective effects. ν(0<ε≪1) is the molecular diffusivity characterizing the Brownian motion, and $b_{1},\;b_{2}$ are convection coefficients. $u_{0}\left(x\right)$ and ψ(x,t) are known functions.

This equation can be considered as the linearized version of the two-dimensional Navier–Stokes equations in vorticity formulation with viscosity ν=1Re (Re is the Reynolds number). When φ(u(x,t))=u(x,t), the equation becomes the classical convection–diffusion equation. The convection–diffusion equation is a fundamental model in fluid dynamics, heat transfer, and mass transport phenomena. It describes the combined effects of convection (transport due to fluid motion) and diffusion (spreading due to concentration gradients) on the evolution of a scalar quantity, such as temperature or concentration. The equation is widely used in various fields, including environmental science, chemical engineering, and materials science. This equation can be considered to be the linearized version of the two-dimensional Navier–Stokes equations in vorticity formulation with viscosity ν=1Re (Re is the Reynolds number). Although multiple-relaxation-time (MRT) and entropic collision operators have been shown to improve numerical stability and robustness for the full Navier–Stokes equations, particularly at high Reynolds numbers, our current work focuses on the BGK model applied to this linearized system as a foundational step. The BGK framework allows for a clear demonstration and validation of the proposed lattice Boltzmann method with an amending function. We acknowledge that incorporating MRT and entropic collision operators could further enhance stability and adaptability, and these extensions are planned for future work once the current model’s effectiveness is fully established. This staged approach ensures the methodological soundness and clarity before advancing to more complex collision models and nonlinear systems.

In order to improve computational efficiency and extend the application of LBM in solving nonlinear partial differential equations, we adopt a D2Q4 discrete velocity model to construct a lattice BGK Boltzmann model with an amending function for Equation (1). By using the Chapman–Enskog expansion [44], The governing evolution equation can be accurately derived from the continuous BGK Boltzmann equation. Comprehensive comparisons between numerical results and analytical solutions are conducted. The simulations demonstrate excellent agreement with the analytical solutions.

The content of this paper is arranged as follows. In Section 2, we introduce the lattice Boltzmann model and derive the governing equation. In Section 3, we present numerical experiments to validate the accuracy and reliability of the proposed model. Finally, we summarize our findings in Section 4.

## 2. Lattice Boltzmann Model

**Figure 1:**
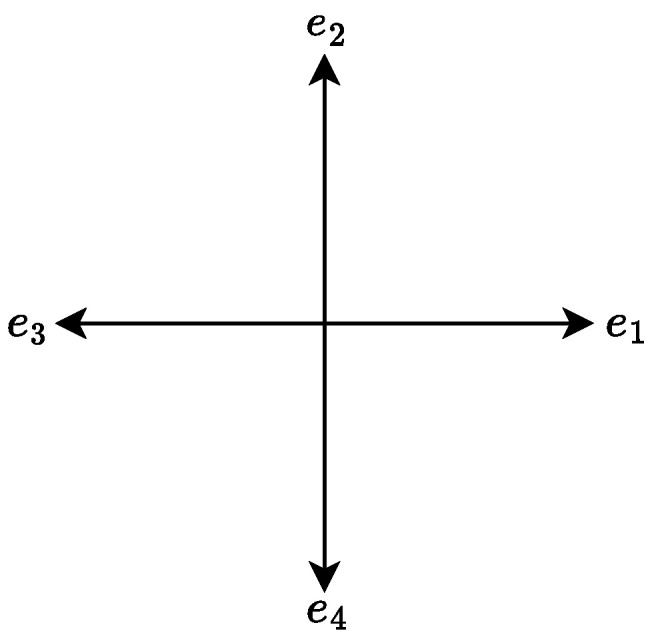
Discrete velocity directions of the D2Q4 lattice scheme.

The lattice Boltzmann model employed in this study is the standard lattice Bhatnagar–Gross–Krook (LBGK) model based on the D2Q4 velocity set. The discrete velocity model is shown in Figure 1, and the D2Q4 lattice consists of four discrete velocities, each aligned with the Cartesian axes. The corresponding velocity vectors $e_{i}$ for i=1,…,4 are defined as follows:

$$\left[e_{1},e_{2},e_{3},e_{4}\right]=\left[\begin{array}{cccc} 1 & 0 & -1 & 0\\ 0 & 1 & 0 & -1 \end{array}\right].$$

The evolution of the particle distribution function $f_{i}$ with an amending source term $S_{i}$ is governed by the following LBGK equation:

(2) $$f_{i}\left(x+c\varepsilon e_{i},t+\varepsilon^{2}T\right)-f_{i}\left(x\right)=-\frac{1}{\tau }\left[f_{i}\left(x,t\right)-f_{i}^{\left(0\right)}\left(x,t\right)\right]+\varepsilon S_{i}\left(x,t\right),$$

where $f_{i}\left(x,t\right)$ and $f_{i}^{\left(0\right)}\left(x,t\right)$ are defined as distribution function and equilibrium distribution function, respectively, $\varepsilon S_{i}\left(x,t\right)$ is an amending function. Here, *c* is the lattice characteristic speed relating spatial and temporal scales via the lattice CFL condition cε=h. The parameter ε is a small expansion parameter related to the Knudsen number, *h* is the spatial step size, and *T* is a characteristic timescale satisfying the diffusive scaling $\Delta t=\varepsilon^{2}T$. The dimensionless relaxation time τ controls the rate of relaxation towards equilibrium and must satisfy the stability condition τ>0.5 [45].

The macroscopic variable u can be defined as the zeroth moment of the distribution function:

(3) $$\begin{array}{c} u\left(x,t\right)=\sum_{i}f_{i}\left(x,t\right), \end{array}$$

and the equilibrium distribution function $f_{i}^{\left(0\right)}\left(x,t\right)$ should meet the following conservation laws:

(4) $$\begin{array}{c} \sum_{i}f_{i}\left(x,t\right)=\sum_{i}f_{i}^{\left(0\right)}\left(x,t\right). \end{array}$$

Then, through choosing appropriate local equilibrium distribution, we can retrieve the corresponding macroscopic equation correctly.

Indeed, applying Taylor expansion to the left hand of Equation (2) and retaining terms up to $O(\varepsilon^{3})$, we can get

(5) $$\begin{array}{ccc} & & \varepsilon^{2}T\frac{\partial f_{i}\left(x,t\right)}{\partial t}+c\varepsilon e_{i}\cdot \nabla f_{i}\left(x,t\right)+\frac{1}{2}c^{2}\Delta t^{2}{\left(e_{i}\cdot \nabla \right)}^{2}f_{i}\left(x,t\right)+O\left(\varepsilon^{3}\right)\\ & = & -\frac{1}{\tau }\left[f_{i}\left(x,t\right)-f_{i}^{\left(0\right)}\left(x,t\right)\right]+\varepsilon S_{i}\left(x,t\right). \end{array}$$

To derive the macroscopic equation Equation (1), the Chapman–Enskog expansion is applied:

(6) $$f_{i}\left(x,t\right)=f_{i}^{\left(0\right)}\left(x,t\right)+\varepsilon f_{i}^{\left(1\right)}\left(x,t\right)+\varepsilon^{2}f_{i}^{\left(2\right)}\left(x,t\right)+O\left(\varepsilon^{3}\right).$$

The Knudsen number ε is defined as follows:

(7) $$\varepsilon =\frac{\ell }{L},$$

where *ℓ* is the mean free path, and *L* is the characteristic length, which can be taken as the time step Δt. And $f_{i}^{\left(k\right)}\left(x,t\right)$ (k=1,2,⋯) represent higher-order corrections associated with nonequilibrium effects at different time scales, which satisfy the solvability conditions:

(8) $$\sum_{i}f_{i}^{\left(k\right)}\left(x,t\right)=0,\;\;\;\left(k=1,2,\cdots \right).$$

Substituting Equation (6) into Equation (5), we obtain a series of lattice Boltzmann equations in different timescales:

$O\left(\varepsilon \right)$:

(9) $$ce_{i}\cdot \nabla f_{i}^{\left(0\right)}\left(x,t\right)-S_{i}\left(x,t\right)=-\frac{1}{\tau }f_{i}^{\left(1\right)}\left(x,t\right);$$

$O\left(\varepsilon^{2}\right)$:

(10) $$\frac{\partial f_{i}^{\left(0\right)}\left(x,t\right)}{\partial t}+\frac{c}{T}e_{i}\cdot \nabla f_{i}^{\left(1\right)}\left(x,t\right)+\frac{c^{2}}{2T}{\left(e_{i}\cdot \nabla \right)}^{2}f_{i}^{\left(0\right)}\left(x,t\right)=-\frac{1}{\tau T}f_{i}^{\left(2\right)}\left(x,t\right).$$

Substituting Equation (9) into Equation (10), we get

(11) $$\frac{\partial f_{i}^{\left(0\right)}\left(x,t\right)}{\partial t}+\frac{\tau c}{T}e_{i}\cdot \nabla S_{i}\left(x,t\right)-\frac{c^{2}}{T}\left(\tau -\frac{1}{2}\right){\left(e_{i}\cdot \nabla \right)}^{2}f_{i}^{\left(0\right)}\left(x,t\right)=-\frac{1}{\tau T}f_{i}^{\left(2\right)}\left(x,t\right).$$

Summing the two sides of Equation (11) with respect to *i* and using Equations (3) and (8), we can obtain

(12) $$\frac{\partial u(x,t)}{\partial t}+\frac{\tau c}{T}\sum_{i}\left(e_{i}\cdot \nabla S_{i}\left(x,t\right)\right)-\frac{c^{2}}{T}\left(\tau -\frac{1}{2}\right)\sum_{i}{\left(e_{i}\cdot \nabla \right)}^{2}f_{i}^{\left(0\right)}\left(x,t\right)=0.$$

We select the local equilibrium distribution functions $f_{i}^{\left(0\right)}\left(x,t\right)$ to satisfy the following:

(13) $$f_{i}^{\left(0\right)}\left(x,t\right)=\frac{1}{4}u\left(x,t\right),$$

and define the amending functions $S_{i}\left(x,t\right)$ as follows:

(14) $$S_{i}\left(x,t\right)=\left\{\begin{array}{c} \eta_{1}\varphi \left(u\left(x,t\right)\right),\;i=1,\\ \eta_{2}\varphi \left(u\left(x,t\right)\right),\;i=2,\\ -\eta_{1}\varphi \left(u\left(x,t\right)\right),\;i=3,\\ -\eta_{2}\varphi \left(u\left(x,t\right)\right),\;i=4, \end{array}\right.$$

where $\eta_{1}$ and $\eta_{2}$ are some constants to be determined. The structure of the amending function $S_{i}$ ensures isotropy and consistency with the target convection term, while allowing for flexible tuning via the parameters $\eta_{1}$ and $\eta_{2}$.

Then, we get

(15) $$\begin{array}{ccc} \sum_{i}\left(e_{i}\cdot \nabla S_{i}\left(x,t\right)\right) & = & \left(\eta_{1}e_{1}+\eta_{2}e_{2}-\eta_{1}e_{3}-\eta_{2}e_{4}\right)\cdot \nabla \varphi \left(u\left(x,t\right)\right)\\ & = & 2\eta_{1}\frac{\partial \varphi \left(u\left(x,t\right)\right)}{\partial x}+2\eta_{2}\frac{\partial \varphi \left(u\left(x,t\right)\right)}{\partial y}, \end{array}$$

and

(16) $$\begin{array}{ccc} \sum_{i}{\left(e_{i}\cdot \nabla \right)}^{2}f_{i}^{\left(0\right)}\left(x,t\right) & = & \frac{1}{4}\sum_{i}{\left(e_{i}\cdot \nabla \right)}^{2}u\left(x,t\right)\\ & = & \frac{1}{2}\left(\frac{\partial^{2}u\left(x,t\right)}{\partial x^{2}}+\frac{\partial^{2}u\left(x,t\right)}{\partial y^{2}}\right). \end{array}$$

Substituting Equations (15) and (16) into Equation (12), we can obtain

(17) $$\begin{array}{c} \frac{\partial u(x,t)}{\partial t}+\frac{2\tau c}{T}\left(\eta_{1}\frac{\partial \varphi (u(x,t))}{\partial x}+\eta_{2}\frac{\partial \varphi (u(x,t))}{\partial y}\right)\\ -\frac{c^{2}}{2T}\left(\tau -\frac{1}{2}\right)\left(\frac{\partial^{2}u\left(x,t\right)}{\partial x^{2}}+\frac{\partial^{2}u\left(x,t\right)}{\partial y^{2}}\right)=0. \end{array}$$

To recover Equation (1), just let

(18) $$\left\{\begin{array}{l} \frac{2\tau c}{T}\eta_{1}=b_{1},\;\\ \frac{2\tau c}{T}\eta_{2}=b_{2},\;\\ \frac{c^{2}}{2T}\left(\tau -\frac{1}{2}\right)=\nu . \end{array}\right.$$

In the computational process, we can let

(19) $$\left\{\begin{array}{c} \Delta x=\Delta y=c\varepsilon =h,\;\\ \Delta t=\varepsilon^{2}T, \end{array}\right.$$

then

(20) $$\left\{\begin{array}{l} \tau =\frac{1}{2}+\frac{2\nu \Delta t}{h^{2}},\;\\ \eta_{1}\varepsilon =\frac{b_{1}}{2\tau }\frac{\Delta t}{h},\;\\ \eta_{2}\varepsilon =\frac{b_{2}}{2\tau }\frac{\Delta t}{h}, \end{array}\right.$$

where $b_{1},b_{2}$ and ν are known parameters of the problem.

With the above choice of parameters, the proposed lattice Boltzmann BGK model, equipped with an amending function, successfully recovers the general form of a two-dimensional nonlinear convection–diffusion equation:

$$\frac{\partial u}{\partial t}+b_{1}\frac{\partial \varphi (u)}{\partial x}+b_{2}\frac{\partial \varphi (u)}{\partial y}=\nu \left(\frac{\partial^{2}u}{\partial x^{2}}+\frac{\partial^{2}u}{\partial y^{2}}\right).$$

This formulation offers a flexible and consistent framework for simulating a broad class of second-order nonlinear partial differential equations, with tunable convective and diffusive components. The incorporation of the amending function $S_{i}$ enables accurate recovery of macroscopic dynamics beyond the scope of standard equilibrium-based LBM approaches.

In summary, the proposed mesoscopic lattice Boltzmann BGK model, built upon the D2Q4 velocity lattice and augmented with analytically derived equilibrium and correction distribution functions, satisfies the required kinetic moment constraints and recovers the target macroscopic equation through Chapman–Enskog analysis in the diffusive limit. This theoretical foundation paves the way for the numerical experiments in the next section, where the model’s accuracy and robustness are systematically validated using benchmark problems with known exact solutions.

## 3. Numerical Simulation

In this section, to test the above model, numerical simulations of SONPDE are performed. The distribution function $f_{i}\left(x,t\right)$ is initialized by setting to equal $f_{i}^{\left(0\right)}\left(x,t\right)$ for all nodes at t=0. And the macroscopic variable u(x,t) in Equation (1) is initialized by initial condition. The non-equilibrium extrapolation scheme proposed by Guo et al. (2002) is used for implementing boundary conditions [46]. To quantitatively assess the numerical accuracy of our proposed model, three different error metrics (MAXE, MAE, GRE) are defined, where $u(x_{i},y_{j},t)$ and $u^{*}\left(x_{i},y_{j},t\right)$ represent the numerical and exact solutions, respectively, with $N_{x}$ and $N_{y}$ denoting the number of grid points in the *x* and *y* directions. All summations are performed over the entire computational domain. In the following numerical simulation, unless otherwise specified, the evolution time *t* is defined as t=nΔt.

The maximum absolute error (MAXE) is defined as follows:

(21) $$\mathrm{MAXE}=\max_{i,j}\left|u\left(x_{i},y_{j},t\right)-u^{*}\left(x_{i},y_{j},t\right)\right|.$$

The mean absolute error (MAE) is defined as follows:

(22) $$\mathrm{MAE}=\sum_{i=1}^{N_{x}}\sum_{j=1}^{N_{y}}\frac{\left|u\left(x_{i},y_{j},t\right)-u^{*}\left(x_{i},y_{j},t\right)\right|}{N_{x}N_{y}}.$$

The global relative error (GRE) is defined as follows:

(23) $$\mathrm{GRE}=\frac{\sum_{i=1}^{N_{x}}\sum_{j=1}^{N_{y}}\left|u\left(x_{i},y_{j},t\right)-u^{*}\left(x_{i},y_{j},t\right)\right|}{\sum_{i=1}^{N_{x}}\sum_{j=1}^{N_{y}}\left|u^{*}\left(x_{i},y_{j},t\right)\right|}.$$

**Example** **1.** *Consider the following convection–diffusion equation in the region 0≤x≤30, 0≤y≤30:*

$$\begin{array}{c} \frac{\partial u}{\partial t}+b_{1}\frac{\partial u}{\partial x}+b_{2}\frac{\partial u}{\partial y}=\nu \left(\frac{\partial^{2}u}{\partial x^{2}}+\frac{\partial^{2}u}{\partial y^{2}}\right), \end{array}$$

*the initial and boundary conditions are given below:*

$$\left\{\begin{array}{c} u\left(x,y,0\right)=\rho_{0}\exp\left[\frac{b}{2\nu }\left(x+y\right)\right]\sin\frac{\pi x}{l_{x}}\sin\frac{\pi y}{l_{y}},\\ u\left(x,0,t\right)=u\left(x,l_{y},t\right)=u\left(0,y,t\right)=u\left(l_{x},y,t\right)=0. \end{array}\right.$$

*The corresponding exact solution for this instance is given as*

$$u\left(x,y,t\right)=\exp\left[-2\beta t+\frac{b}{2\nu }\left(x+y\right)\right]\sin\frac{\pi x}{30}\sin\frac{\pi y}{30},$$

*where*

$$\beta =\frac{\nu }{4}\left[4{\left(\frac{\pi }{30}\right)}^{2}+{\left(\frac{b}{\nu }\right)}^{2}\right].$$

**Figure 2:**
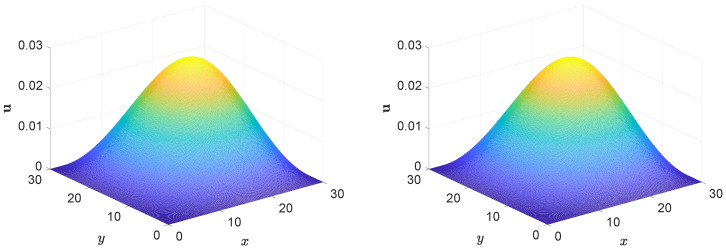
Comparison of the numerical (**left**) and exact (**right**) solutions of the convection–diffusion equation at t=5000.

In the simulation, we use Δx=0.1, Δt=0.1, $b_{1}=b_{2}=0.0004$, and ν=0.004. The simulation results of Figure 2 demonstrate that the numerical solution behaves in a similar pattern to the analytical one across the domain builds confidence in the correctness and reliability of the numerical approach. It reveals the global behavior of the system, showing its structure and characteristics after long-term evolution. This visualization aids in understanding the dynamic behavior of the complex system.

**Figure 3:**
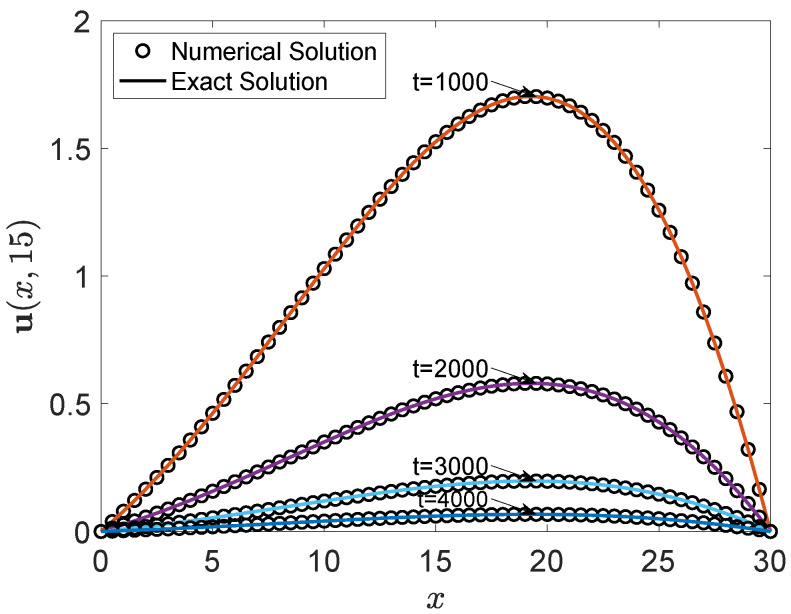
Comparison between the numerical and exact solution of u(x,t) along the line y=15 at multiple time instances.

Additionally, we present the two-dimensional visual comparison of profiles at y=15 at specific times in Figure 3. The two-dimensional profile comparison at y=15 at specific times in Figure 3 provides detailed local evolution information, further validating the accuracy of the numerical results against the exact solutions. The numerical results exhibit a good match with the exact solutions, confirming the accuracy of the method. The comparison shows that the numerical solution captures the essential features of the exact solution, including its spatial distribution and temporal evolution.

**Figure 4:**
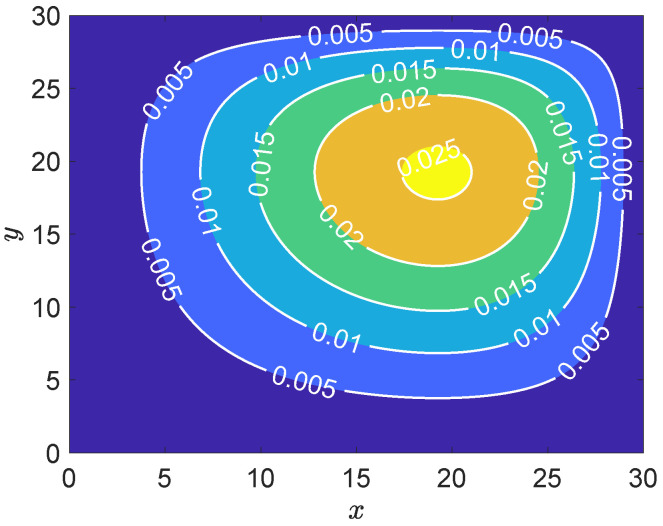
Contour plot of numerical solution for the scalar field u(x,t) at t=5000.

Figure 4 presents the contour plot of the numerical solution to the two-dimensional convection–diffusion equation. It is observed that the solution exhibits a smooth spatial distribution, with values ranging approximately from 0.005 to 0.025. The solution reaches relatively higher values near the central region, indicating the presence of a source term or boundary-driven high concentration area. A clear gradient along a principal direction, possibly from the lower left to the upper right, suggests a dominant convective flow in that direction. This behavior is typical in convection–diffusion problems, where the solution is advected along the flow direction while being smoothed by diffusion. Overall, the contour lines are well-behaved and uniformly distributed without numerical oscillations or non-physical artifacts. This indicates that the adopted numerical scheme possesses good stability and conservativeness, effectively capturing the combined effects of convection and diffusion.

Furthermore, the MAXE, MAE, and GRE for solutions of Example 1 at different times are summarized in Table 1. The table demonstrates the decreasing trend of errors as time progresses, indicating the stability and accuracy of the numerical method. Specifically, the MAXE reduces from $1.5759\times 10^{-4}$ at t=1000 to $1.5427\times 10^{-6}$ at t=5000, showcasing a significant improvement in precision over time. Similarly, the MAE and GRE also exhibit consistent reductions, confirming the reliability of the proposed lattice Boltzmann model for solving the convection–diffusion equation. These results highlight the effectiveness of the numerical scheme in capturing the dynamics of the system with high accuracy.

To verify the spatial accuracy of the proposed lattice Boltzmann model, we use this example for simulation. The simulation is conducted using four different grid resolutions: 150×150, 300×300, and 600×600. The parameters are set as $b_{1}=b_{2}=0.0004$, ν=0.004, and the simulation is run until t=1000. The GRE and the corresponding convergence rate are presented in Table 2. The convergence rate is calculated using the following formula:

$$\mathrm{Order}=\frac{\log(E_{1}/E_{2})}{\log(h_{1}/h_{2})},$$

where $E_{1}$ and $E_{2}$ are the MAXE corresponding to grid spacings $h_{1}$ and $h_{2}$, respectively. As shown in Table 2, the GRE decreases as the grid is refined, and the convergence rate approaches 2, which is consistent with the theoretical second-order accuracy of the LBM.

**Example** **2.** *Consider the following the convection–diffusion equation in the region 0≤x≤2, 0≤y≤2 with the initial and boundary conditions [47]:*

$$\begin{array}{c} \frac{\partial u}{\partial t}+b_{1}\frac{\partial u}{\partial x}+b_{2}\frac{\partial u}{\partial y}=\nu \left(\frac{\partial^{2}u}{\partial x^{2}}+\frac{\partial^{2}u}{\partial y^{2}}\right). \end{array}$$

*The initial conditions are given below:*

$$\begin{array}{c} u\left(x,y,0\right)=\exp\left[-\frac{{\left(x-0.5\right)}^{2}+{\left(y-0.5\right)}^{2}}{\nu }\right]. \end{array}$$

*The corresponding exact solution for this instance is given that*

$$u\left(x,y,t\right)=\frac{1}{4t+1}\exp\left[-\frac{{\left(x-b_{1}t-0.5\right)}^{2}+{\left(y-b_{2}t-0.5\right)}^{2}}{\nu (4t+1)}\right].$$

*The boundary conditions can be extracted from the exact solution.*

**Figure 5:**
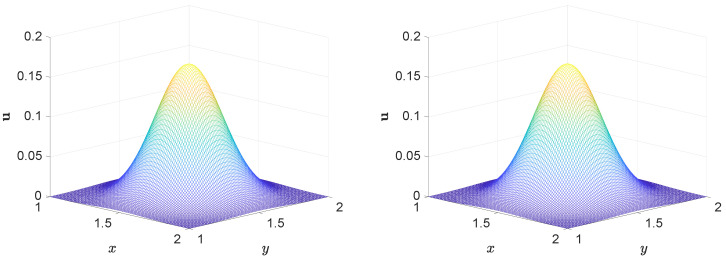
Comparison of the numerical (**left**) and exact (**right**) solutions of the convection–diffusion equation at t=1.25.

In the simulation, we use Δx=0.01, Δt=0.0001, $b_{1}=b_{2}=0.8$, and ν=0.01. The simulation results in Figure 5 show a strong agreement between the numerical and analytical solutions over the entire computational domain. The solution surfaces exhibit similar structural patterns, demonstrating that the numerical scheme accurately captures the essential features of the exact solution. This close match not only verifies the correctness of the proposed method but also reflects the method’s capability in resolving the long-term dynamic behavior of the system. The three-dimensional visualization further enhances our understanding of the system’s global evolution.

**Figure 6:**
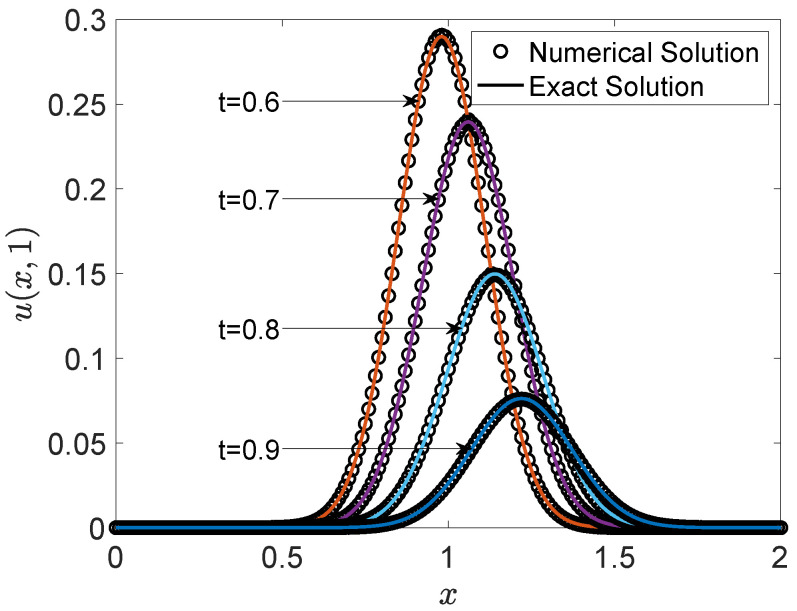
Comparison between the numerical and exact solutions along the line y=1 at multiple time instances, highlighting the temporal evolution of the scalar field u(x,t).

In addition, we present the two-dimensional visual comparison of profiles at y=1 at specific times in Figure 6. The comparison provides detailed insights into the local evolution of the solution, allowing for a more precise evaluation of the numerical method’s accuracy. The numerical profiles are in good agreement with the corresponding exact solutions, clearly reproducing both the spatial structure and the temporal development of the exact solution. This consistency further confirms that the proposed numerical scheme effectively captures the key dynamic features of the system.

**Figure 7:**
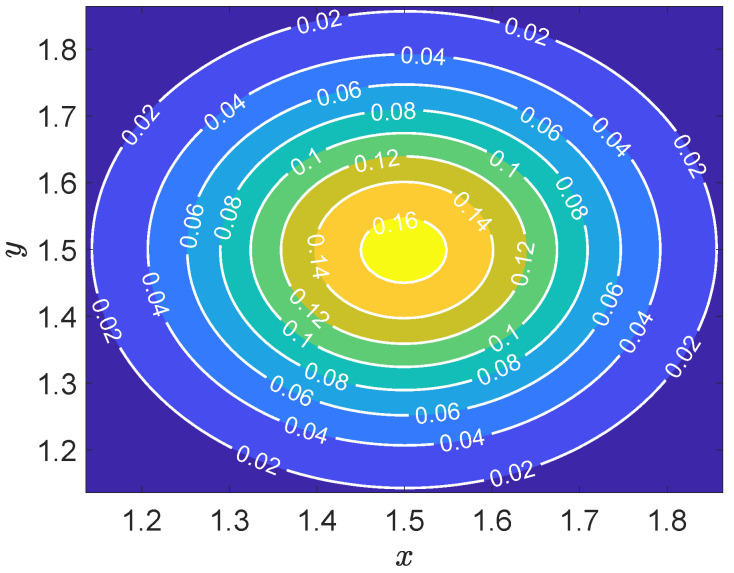
Contour plot of the numerical solution for the scalar field u(x,t) at t=1.25.

Figure 7 presents the contour plot of the numerical solution to the two-dimensional convection–diffusion equation. The solution exhibits a smooth and continuous spatial distribution, with values ranging approximately from 0.02 to 0.16. A prominent high-concentration region is observed near the upper central part of the domain, likely corresponding to a localized source term or boundary-induced effect. The contour lines reveal a distinct gradient oriented from the lower left toward the upper right, indicative of a dominant convective flow in that direction. This directional behavior is consistent with the expected dynamics in convection–diffusion problems, where convection transports the quantity along a flow path while diffusion smooths out local variations. The uniformly spaced and well-behaved contour lines suggest that the employed numerical scheme is stable and conservative, effectively capturing the interplay between convective and diffusive processes without introducing non-physical artifacts.

Furthermore, the MAXE, MAE, and GRE for solutions of Example 2 at different times are summarized in Table 3. The table shows that the errors decrease as time progresses, indicating the stability and accuracy of the numerical method. Specifically, the MAXE reduces from $1.2698\times 10^{-3}$ at t=0.6 to $7.5822\times 10^{-4}$ at t=1.0, showcasing a significant improvement in precision over time. Similarly, the MAE and GRE also exhibit consistent reductions, confirming the reliability of the proposed lattice Boltzmann model for solving the convection–diffusion equation. These results highlight the effectiveness of the numerical scheme in capturing the dynamics of the system with high accuracy.

To investigate the influence of the relaxation time τ on the numerical accuracy of the proposed LBM model, we conduct an additional test based on the setup of Example 2. The spatial discretization is Δx=Δy=h=0.01, and the time step is fixed at Δt=0.0001. The relaxation time τ is varied by selecting different viscosity coefficients ν. All simulations are performed up to a final time of t=0.5. The errors (MAXE, MAE, GRE) are computed and compared for these different ν values (and corresponding τ values), as shown in Table 4.

**Figure 8:**
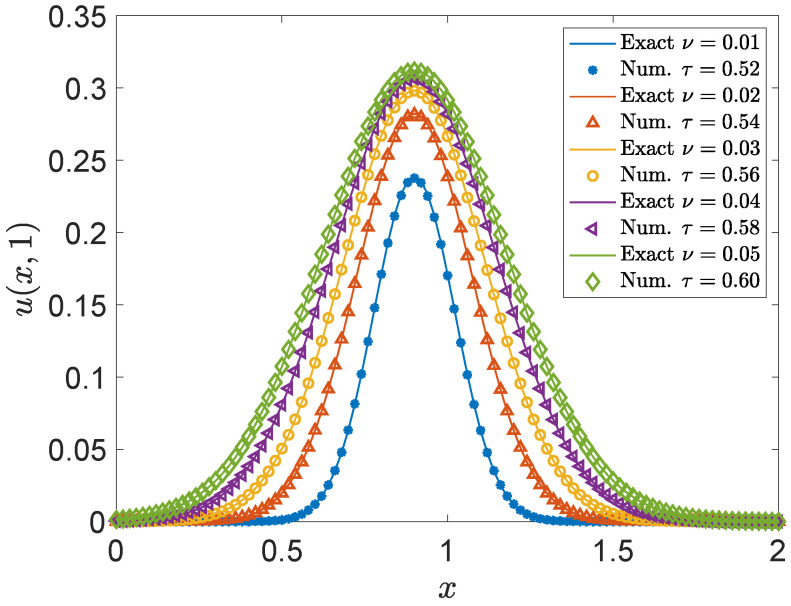
Comparison of numerical solutions and analytical solution at y=1 for different relaxation times τ at time t=0.5. Solid lines represent the analytical solution, while markers indicate numerical results corresponding to various τ values.

The results presented in Table 4 (with illustrative error values) and Figure 8 show how varying the viscosity coefficient ν, and consequently the relaxation time τ, affects numerical accuracy when other parameters like Δt and *h* are fixed. In this setup, as ν increases, τ also increases (from 0.52 to 0.60 in the table). The illustrative error values reveal a clear trend that larger ν (and thus larger τ) correspond to decreased errors for the fixed Δt and *h* considered here. This implies that, for a given discretization, higher physical viscosity ν (leading to larger τ values) may result in improved numerical accuracy, while still maintaining τ>0.5 to ensure stability. It is important to emphasize that ν is an inherent physical property of the system. The numerical parameters Δt and *h* must be selected in coordination with the physical viscosity ν to guarantee that the relaxation time τ remains within a stable and accurate range, balancing computational cost and precision. When the physical ν is very small, causing τ to approach the lower stability limit of 0.5, adjustments to Δt or *h* may be necessary to maintain both stability and accuracy, which often entails a trade-off with computational efficiency.

**Example** **3.** *Consider the following the diffusion equation in the region 0⩽x⩽1,0⩽y⩽1 with initial and boundary conditions [48]:*

$$\frac{\partial u}{\partial t}+b_{1}\frac{\partial u}{\partial x}+b_{2}\frac{\partial u}{\partial y}=\nu \left(\frac{\partial^{2}u}{\partial x^{2}}+\frac{\partial^{2}u}{\partial y^{2}}\right).$$

*The initial and boundary conditions are given below:*

$$\left\{\begin{array}{c} u\left(0,y,t\right)=u\left(1,y,t\right)=u\left(x,0,t\right)=u\left(x,1,t\right)=0,\\ u\left(x,y,0\right)=\sin\left(\pi x\right)\sin\left(\pi y\right). \end{array}\right.$$

*The corresponding exact solution for this instance is given as*

$$u\left(x,y,t\right)=\exp\left(-2\pi^{2}t\right)\sin\left(\pi x\right)\sin\left(\pi y\right).\;$$

**Figure 9:**
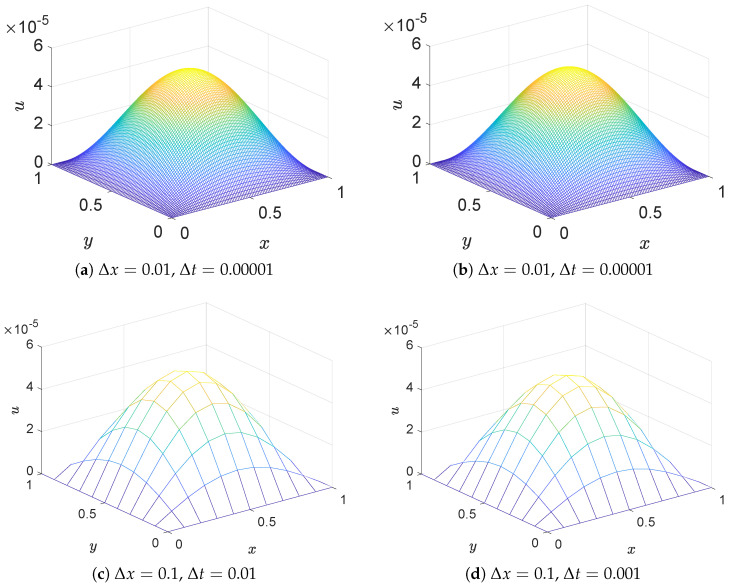
Comparison of the numerical (**left**) and exact (**right**) solutions of the scalar field u(x,0.5) under different grid resolutions.

In this example, we perform a basic sensitivity and stability analysis by simulating the model with two different sets of spatial and temporal resolutions: $\Delta x_{1}=0.01$ and $\Delta x_{2}=0.1$ for the simulation. To satisfy the Courant–Friedrichs–Lewy (CFL) stability condition in numerical simulations, we set $\Delta t_{1}=0.00001$ for the case of $\Delta x_{1}=0.01$, and $\Delta t_{2}=0.001$ for the case of $\Delta x_{2}=0.1$. For both spatial step sizes, we set ν=1.0. This test is designed to evaluate the robustness of the proposed scheme under different discretization parameters. The simulation results indicate that, regardless of the selected spatial and temporal step sizes, the numerical solutions remain in excellent agreement with the exact solutions throughout the time evolution. The simulation results demonstrate that regardless of the values chosen for Δx and Δt, the numerical solutions maintain excellent agreement with the exact solutions during long-term evolution. Similar to previous examples, we employ smaller time steps and spatial steps to ensure high computational accuracy. Figure 9a,c display three-dimensional images of the exact solution at t = 0.5, showing a shape with a high center and low periphery, which presents certain computational challenges due to significant numerical variations. However, comparison between the three-dimensional numerical solution illustrations in Figure 9b,d and the exact solution illustrations reveals a high degree of concordance between numerical and exact solutions, independent of the selected values for Δx and Δt. These results provide preliminary evidence of the reliability and robustness of the proposed model. Further tests involving a wider range of parameters, including relaxation times, will be explored in future work.

**Figure 10:**
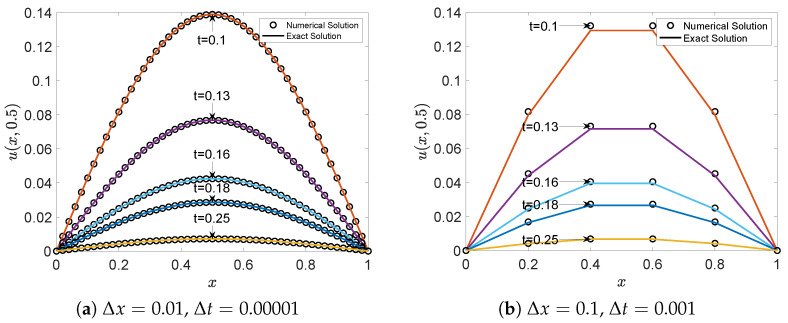
Comparison of numerical and exact solution at the line of y=0.5 at different time instances.

Figure 10a,b present two-dimensional profiles at y=0.5 at different time intervals, providing detailed information about local function variations during the evolution process, further confirming the consistency between numerical and exact solutions. For error analysis, Table 5 display the MAXE, MAE, and GRE for this example. The numerical values in Table 5 indicate that errors remain minimal over extended time periods, validating the effectiveness and reliability of this model.

**Example** **4.** *Consider the following unsteady convection–diffusion equation with initial and boundary conditions in the domain Ω={(x,y,t)|0≤x≤L,0≤y≤L,0≤t≤T} [49]:*

$$\frac{\partial u}{\partial t}+\beta_{x}\frac{\partial u}{\partial x}+\beta_{y}\frac{\partial u}{\partial y}=\alpha_{x}\frac{\partial^{2}u}{\partial x^{2}}+\alpha_{y}\frac{\partial^{2}u}{\partial y^{2}}.$$

*The initial condition is given by the following:*

$$u\left(x,y,0\right)=a(\exp\left(-c_{x}x\right)+\exp\left(-c_{y}y\right)),$$

*where*

$$c_{x}=\frac{-\beta_{x}\pm \sqrt{\beta_{x}^{2}+4b\alpha_{x}}}{2\alpha_{x}}>0,\;c_{y}=\frac{-\beta_{y}\pm \sqrt{\beta_{y}^{2}+4b\alpha_{y}}}{2\alpha_{y}}>0.$$

*It can be verified that the exact solution is the following*

$$u\left(x,y,t\right)=a\exp\left(bt\right)(\exp\left(-c_{x}\cdot x\right)+\exp\left(-c_{y}\cdot y\right)).$$

*The boundary conditions can be easily derived from the exact solution.*

**Figure 11:**
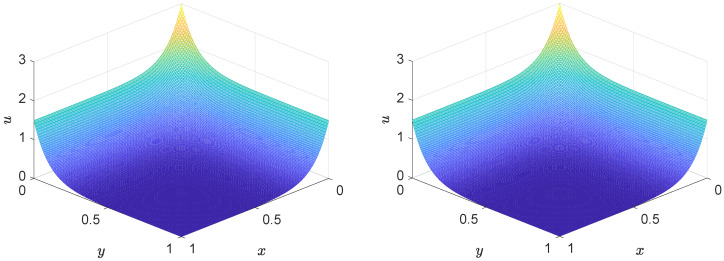
Comparison of the numerical (**left**) and exact (**right**) solutions of the unsteady convection–diffusion equation at t=4.0.

In the simulation, L=1.0, T=4.0, Δx=0.005, Δt=0.0001, $b_{1}=b_{2}=-1.0$, a=1.0, b=0.1, and ν=0.1. The three-dimensional surface plots of the two-dimensional numerical solutions at t=4.0 are illustrated in Figure 11, which provide a clearer visualization of spatial variations over the 2D domain. A close agreement between the numerical solution (left) and the exact solution (right) is observed, demonstrating the high accuracy and reliability of the proposed lattice Boltzmann model. Both solutions exhibit smooth, symmetric surfaces characterized by a pronounced peak near the center and a gradual decay towards the domain boundaries along both the *x*- and *y*-axes. The numerical method successfully reproduces the essential dynamics of the convection–diffusion system, capturing both the spatial smoothness and the outward decay in solution values. These results confirm the model’s capability to accurately resolve the combined effects of convection and diffusion in unsteady transport processes.

**Figure 12:**
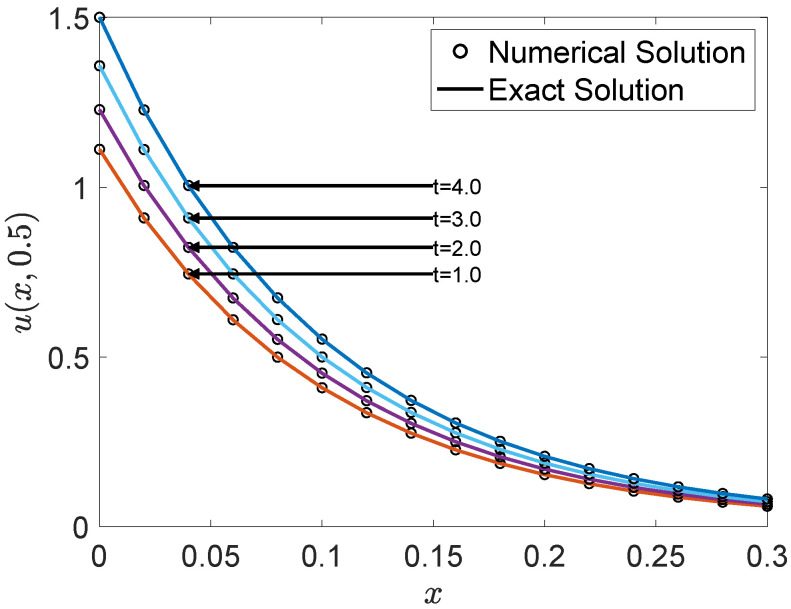
Comparison between the numerical and exact solutions along the line y=0.5 at multiple time instances.

Furthermore, Figure 12 provides a two-dimensional comparison of the numerical and exact solutions along the line y=0.5 at multiple time instances. The numerical profiles align well with the exact solutions, confirming the model’s ability to accurately simulate the temporal evolution of the scalar field u(x,t).

**Figure 13:**
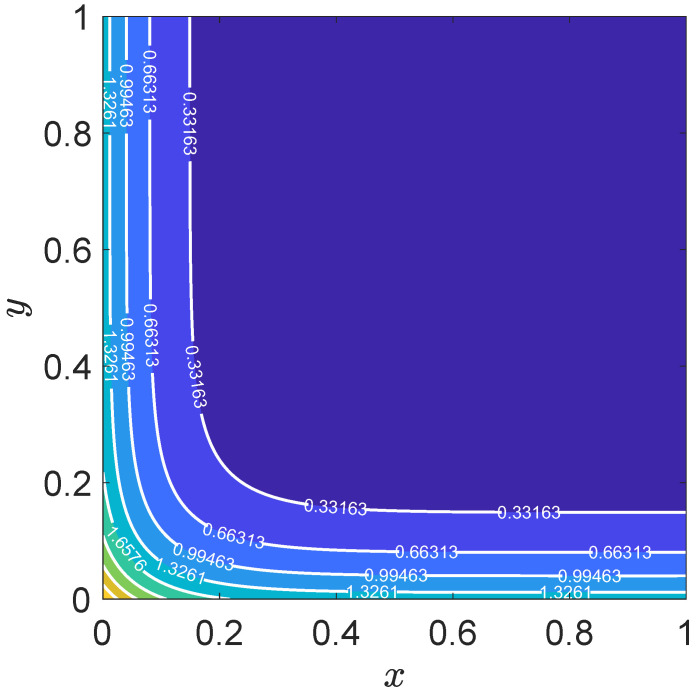
Contour plot of the numerical solution for the scalar field u(x,t) at t=4.0.

The contour plot in Figure 13 further illustrates the spatial distribution of the numerical solution at t=4.0. The contours are smooth and well-behaved, indicating the stability of the numerical scheme. The results highlight the model’s capability to handle complex boundary conditions and accurately resolve the interplay between convection and diffusion.

The MAXE, MAE, and GRE values in Table 6 demonstrate the model’s high accuracy over time. Due to the system’s unsteadiness, the MAXE increases slightly from $8.1804\times 10^{-4}$ at t=0.5 to $1.1607\times 10^{-3}$ at t=4.0, indicating that the numerical solution still remains close to the exact solution throughout the simulation. Similarly, the MAE values reflect the overall consistency of the numerical results. The GRE values remain stable around $6.7948\times 10^{-4}$, further confirming the robustness and reliability of the proposed method for solving unsteady convection–diffusion equations. These results highlight the effectiveness of the lattice Boltzmann model in accurately capturing the dynamics of the system over time.

## 4. Conclusions

In this study, a lattice BGK model incorporating an amending function was developed to solve two-dimensional second-order nonlinear partial differential Equation (SONPDE). The Chapman–Enskog expansion rigorously demonstrated the model’s ability to recover the target macroscopic equations in the continuous limit, ensuring theoretical consistency between the mesoscopic framework and the macroscopic physics. A D2Q4 discrete velocity set was employed to construct the model, enabling efficient computation and clear derivation of the moment constraints for both equilibrium and correction terms. Extensive numerical simulations were conducted on multiple benchmark problems. The proposed model exhibited excellent agreement with exact solutions, confirming its accuracy, stability, and robustness across a variety of parameter regimes and initial conditions. Furthermore, the model demonstrated strong performance over long-term evolutions, showcasing its capability to capture essential convection–diffusion dynamics with high precision. The simplicity of the D2Q4 lattice and the modularity of the correction scheme make the approach computationally attractive for large-scale simulations and parallel implementations. This study establishes a solid foundation for further kinetic-based modeling of nonlinear PDEs in two dimensions.

Despite these promising results, the current model also faces some limitations. First, the use of the D2Q4 lattice restricts the method to relatively simple geometries and structured grids; its application to irregular or complex domains may require non-trivial modifications or hybrid approaches. Second, the extension to three-dimensional problems is not straightforward, as it demands careful design of the discrete velocity set and appropriate correction mechanisms to maintain accuracy and stability. Furthermore, the current formulation is tailored for scalar equations; extending it to coupled systems or vector-valued fields introduces additional modeling challenges that merit future investigation.

Future research will aim to (1) extend the current framework to nonlinear, coupled, higher-order or more general partial differential equations, such as reaction–diffusion systems, heat and mass transfer equations with variable coefficients, and fluid–structure interaction problems; (2) integrate multiple relaxation time (MRT) and entropic collision operators to enhance numerical stability and adaptability to high Reynolds number flows; (3) incorporate thermodynamic and hydrodynamic nonequilibrium effects to simulate strongly coupled or multiphysics processes such as reactive flows and multiphase transport; and (4) explore coupling with machine learning techniques for adaptive parameter tuning and model acceleration. These directions will not only expand the applicability of the proposed model but also facilitate its use in more realistic and complex scientific and engineering problems.

## Figures and Tables

**Table 1:** The MAXE, MAE, and GRE for solutions of Example 1 at different times.

*t*	1000	2000	3000	4000	5000
MAXE	$1.5759\times 10^{-4}$	$5.3258\times 10^{-5}$	$1.7709\times 10^{-5}$	$5.6383\times 10^{-6}$	$1.5427\times 10^{-6}$
MAE	$4.4641\times 10^{-5}$	$1.5591\times 10^{-5}$	$5.3187\times 10^{-6}$	$1.8569\times 10^{-6}$	$6.6583\times 10^{-7}$
GRE	$1.5759\times 10^{-4}$	$5.5327\times 10^{-5}$	$5.4637\times 10^{-5}$	$1.0338\times 10^{-5}$	$6.1527\times 10^{-5}$

**Table 2:** GRE and convergence rate for Example 1 at t=1000 with different grid resolutions.

Grid Size	GRE	Convergence Rate
150×150	$1.8486\times 10^{-4}$	-
300×300	$4.3617\times 10^{-5}$	2.0835
600×600	$6.0608\times 10^{-6}$	2.8473

**Table 3:** The MAXE, MAE, and the GRE for solutions of Example 2 at different times.

*t*	0.6	0.7	0.8	0.9	1.0
MAXE	$1.2698\times 10^{-3}$	$1.0966\times 10^{-3}$	$9.6093\times 10^{-4}$	$8.5047\times 10^{-4}$	$7.5822\times 10^{-4}$
MAE	$3.1070\times 10^{-5}$	$3.0495\times 10^{-5}$	$2.9859\times 10^{-5}$	$2.9206\times 10^{-5}$	$2.8556\times 10^{-5}$
GRE	$3.4503\times 10^{-3}$	$3.3882\times 10^{-3}$	$3.3194\times 10^{-3}$	$3.2484\times 10^{-3}$	$3.1778\times 10^{-3}$

**Table 4:** Error comparison at t=0.5 for different values of ν and corresponding relaxation time τ.

ν	τ	MAXE	MAE	GRE
0.01	0.5200	$1.4850\times 10^{-3}$	$3.1510\times 10^{-4}$	$3.4973\times 10^{-3}$
0.02	0.5400	$5.6425\times 10^{-4}$	$2.2809\times 10^{-5}$	$1.2572\times 10^{-3}$
0.03	0.5600	$3.1790\times 10^{-4}$	$1.8696\times 10^{-5}$	$6.8435\times 10^{-4}$
0.04	0.5800	$2.0857\times 10^{-4}$	$1.6480\times 10^{-5}$	$4.5091\times 10^{-4}$
0.05	0.6000	$1.4775\times 10^{-4}$	$1.5929\times 10^{-5}$	$3.4398\times 10^{-4}$

**Table 5:** MAXE, MAE, and GRE for solutions of Example 3 at different times.

*t*	0.10	0.13	0.16	0.18	0.25
Δx=0.01 and y=0.5					
MAXE	$1.8289\times 10^{-5}$	$8.4363\times 10^{-6}$	$3.7363\times 10^{-6}$	$2.1058\times 10^{-6}$	$1.6160\times 10^{-7}$
MAE	$7.5792\times 10^{-6}$	$2.5260\times 10^{-6}$	$1.5820\times 10^{-6}$	$9.0045\times 10^{-7}$	$8.0664\times 10^{-8}$
GRE	$1.3595\times 10^{-4}$	$1.1412\times 10^{-4}$	$9.2302\times 10^{-5}$	$3.0374\times 10^{-4}$	$2.6827\times 10^{-5}$
Δx=0.1 and y=0.5					
MAXE	$2.9103\times 10^{-3}$	$1.6736\times 10^{-3}$	$9.6099\times 10^{-4}$	$6.6338\times 10^{-4}$	$1.8051\times 10^{-4}$
MAE	$1.1591\times 10^{-3}$	$6.6209\times 10^{-4}$	$3.7779\times 10^{-4}$	$2.5976\times 10^{-4}$	$6.9797\times 10^{-5}$
GRE	$2.4519\times 10^{-2}$	$2.5347\times 10^{-2}$	$2.6174\times 10^{-2}$	$2.6725\times 10^{-2}$	$2.8652\times 10^{-2}$

**Table 6:** The MAXE, MAE, and GRE for solutions of Example 4 at different times.

*t*	0.5	1.0	2.0	3.0	4.0
MAXE	$8.1804\times 10^{-4}$	$8.6003\times 10^{-4}$	$9.5033\times 10^{-4}$	$1.0498\times 10^{-3}$	$1.1607\times 10^{-3}$
MAE	$1.5321\times 10^{-4}$	$1.6167\times 10^{-4}$	$1.7868\times 10^{-4}$	$1.9748\times 10^{-4}$	$2.1825\times 10^{-4}$
GRE	$6.7775\times 10^{-4}$	$6.7944\times 10^{-4}$	$6.7948\times 10^{-4}$	$6.7948\times 10^{-4}$	$6.7948\times 10^{-4}$

## Data Availability

The original contributions presented in this study are included in the article. Further inquiries can be directed to the corresponding author.

## References

[1] Debnath L. (2005). Nonlinear Partial Differential Equations for Scientists and Engineers.

[2] Evans L.C. (2022). Partial Differential Equations.

[3] Roubíček T. (2013). Nonlinear Partial Differential Equations with Applications.

[4] Yaghoobi H., Torabi M. (2011). The application of differential transformation method to nonlinear equations arising in heat transfer. Int. Commun. Heat Mass Transf..

[5] Lai H., Ma C. (2011). Lattice Boltzmann model for generalized nonlinear wave equations. Phys. Rev. E.

[6] Krasnow R. (2025). Making partial differential equations accessible to ecologists. Nat. Rev. Biodivers..

[7] Tadmor E. (2012). A review of numerical methods for nonlinear partial differential equations. Bull. Am. Math. Soc..

[8] Feng X., Glowinski R., Neilan M. (2013). Recent developments in numerical methods for fully nonlinear second order partial differential equations. SIAM Rev..

[9] Benzi R., Succi S., Vergassola M. (1992). The lattice Boltzmann equation: Theory and applications. Phys. Rep..

[10] Succi S. (2001). The Lattice Boltzmann Equation: For Fluid Dynamics and Beyond.

[11] Aidun C.K., Clausen J.R. (2010). Lattice Boltzmann method for complex flows. Annu. Rev. Fluid Mech..

[12] Xu A., Zhang G., Gan Y., Chen F., Yu X. (2012). Lattice Boltzmann modeling and simulation of compressible flows. Front. Phys..

[13] Xu A., Zhang D., Gan Y. (2024). Advances in the kinetics of heat and mass transfer in near-continuous complex flows. Front. Phys..

[14] Gan Y., Xu A., Zhang G., Succi S. (2015). Discrete Boltzmann modeling of multiphase flows: Hydrodynamic and thermodynamic non-equilibrium effects. Soft Matter.

[15] Gan Y., Xu A., Zhang G., Zhang Y., Succi S. (2018). Discrete Boltzmann trans-scale modeling of high-speed compressible flows. Phys. Rev. E.

[16] Gan Y., Xu A., Zhang G., Lin C., Lai H., Liu Z. (2019). Nonequilibrium and morphological characterizations of Kelvin–Helmholtz instability in compressible flows. Front. Phys..

[17] Gan Y., Xu A., Lai H., Li W., Sun G., Succi S. (2022). Discrete Boltzmann multi-scale modelling of non-equilibrium multiphase flows. J. Fluid Mech..

[18] Sun G., Gan Y., Xu A., Shi Q. (2024). Droplet coalescence kinetics: Thermodynamic non-equilibrium effects and entropy production mechanism. Phys. Fluids.

[19] Lai H., Lin C., Gan Y., Li D., Chen L. (2023). The influences of acceleration on compressible Rayleigh–Taylor instability with non-equilibrium effects. Comput. Fluids.

[20] Lai H., Li D., Lin C., Chen L., Ye H., Zhu J. (2024). Investigation of effects of initial interface conditions on the two-dimensional single-mode compressible Rayleigh–Taylor instability: Based on the discrete Boltzmann method. Comput. Fluids.

[21] Wolf-Gladrow D.A. (2004). Lattice-Gas Cellular Automata and Lattice Boltzmann Models: An Introduction.

[22] Chen S., Doolen G.D. (1998). Lattice Boltzmann method for fluid flows. Annu. Rev. Fluid Mech..

[23] Guo Z., Shu C. (2013). Lattice Boltzmann Method and Its Application in Engineering.

[24] Wei Y., Yang H., Dou H., Lin Z., Wang Z., Qian Y. (2018). A novel two-dimensional coupled lattice Boltzmann model for thermal incompressible flows. Appl. Math. Comput..

[25] Wei Y., Dou H., Qian Y., Wang Z. (2017). A novel two-dimensional coupled lattice Boltzmann model for incompressible flow in application of turbulence Rayleigh–Taylor instability. Comput. Fluids.

[26] Wang H. (2020). Numerical simulation for (3+1) D solitary wave of extended Zakharov–Kuznetsov equation in dusty plasma based on lattice Boltzmann method. Phys. Lett. A.

[27] Gorakifard M., Salueña C., Cuesta I., Kian Far E. (2022). The meshless local Petrov–Galerkin cumulant lattice Boltzmann method: Strengths and weaknesses in aeroacoustic analysis. Acta Mech..

[28] Boghosian B.M., Dubois F., Lallemand P. (2024). Numerical approximations of a lattice Boltzmann scheme with a family of partial differential equations. Comput. Fluids.

[29] Chen Y., Chai Z., Shi B. (2024). A general fourth-order mesoscopic multiple-relaxation-time lattice Boltzmann model and its macroscopic finite-difference scheme for two-dimensional diffusion equations. J. Comput. Phys..

[30] Chai Z., Shi B., Guo Z. (2016). A multiple-relaxation-time lattice Boltzmann model for general nonlinear anisotropic convection–diffusion equations. J. Sci. Comput..

[31] Wang H., Liu Y., Li X., Chen H. (2024). Numerical simulation for solitary waves of the generalized Zakharov equation based on the lattice Boltzmann method. Mathematics.

[32] Wang H., Chen H., Li T. (2024). Numerical simulation for the wave of the variable coefficient nonlinear Schrödinger equation based on the lattice Boltzmann method. Mathematics.

[33] Liang H., Liu W., Li Y., Wei Y. (2024). A thermal lattice Boltzmann model for evaporating multiphase flows. Phys. Fluids.

[34] Chen Y., Liu X., Chai Z., Shi B. (2025). A Cole–Hopf transformation based fourth-order multiple-relaxation-time lattice Boltzmann model for the coupled Burgers’ equations. J. Sci. Comput..

[35] Chen Y., Liu X., Chai Z., Shi B. (2025). Regularized lattice Boltzmann method based maximum principle and energy stability preserving finite-difference scheme for the Allen-Cahn equation. J. Comput. Phys..

[36] Li Q., Chai Z., Shi B. (2025). Lattice Boltzmann model for a class of viscous wave equation. Adv. Appl. Math. Mech..

[37] Du R., Zhou T., Pang G. (2025). Forward and inverse problem solvers for Reynolds-averaged Navier–Stokes equations with fractional Laplacian. Eng. Anal. Bound. Elem..

[38] Gong H., Zhou T., Shi B., Du R. (2025). Lattice Boltzmann method for surface quasi-geostrophic equations with fractional Laplacian. Appl. Math. Lett..

[39] Bocanegra J.A., Misale M., Borelli D. (2024). A systematic literature review on lattice Boltzmann method applied to acoustics. Eng. Anal. Bound. Elem..

[40] Ginzburg I. (2025). The lattice Boltzmann method with deformable boundary for colonic flow due to segmental circular contractions. Fluids.

[41] Maquart T., Noël R., Courbebaisse G., Navarro L. (2022). Toward a lattice Boltzmann method for solids—Application to static equilibrium of isotropic materials. Appl. Sci..

[42] Hosseini S.A., Boivin P., Thévenin D., Karlin I. (2024). Lattice Boltzmann methods for combustion applications. Prog. Energy Combust. Sci..

[43] Wawrzyniak D., Winter J., Schmidt S., Indinger T., Janßen C.F., Schramm U., Adams N.A. (2025). A quantum algorithm for the lattice-Boltzmann method advection-diffusion equation. Comput. Phys. Commun..

[44] Chapman S., Cowling T.G. (1990). The Mathematical Theory of Non-Uniform Gases: An Account of the Kinetic Theory of Viscosity, Thermal Conduction and Diffusion in Gases.

[45] Sterling J.D., Chen S. (1996). Stability analysis of lattice Boltzmann methods. J. Comput. Phys..

[46] Guo Z., Zheng C., Shi B. (2002). Non-equilibrium extrapolation method for velocity and pressure boundary conditions in the lattice Boltzmann method. Chin. Phys..

[47] Noye B., Tan H. (1989). Finite difference methods for solving the two-dimensional advection–diffusion equation. Int. J. Numer. Methods Fluids.

[48] Bao-Lin Z., Xiu-Min S. (1991). Alternating block explicit-implicit method for the two-dimensional diffusion equation. Int. J. Comput. Math..

[49] Dehghan M., Mohebbi A. (2008). High-order compact boundary value method for the solution of unsteady convection–diffusion problems. Math. Comput. Simul..

